# A lightweight and explainable CNN model for empowering plant disease diagnosis

**DOI:** 10.1038/s41598-025-94083-1

**Published:** 2025-08-21

**Authors:** Chiranjit Pal, Swastik Karmakar, Imon Mukherjee, Partha Pratim Chakrabarti

**Affiliations:** 1 Computer Science & Engineering, Indian Institute of Information Technology, Kalyani, India; 2 Computer Science & Engineering - AI&ML, Heritage Institute of Technology, Kolkata, India; 3 Computer Science & Engineering, Indian Institute of Technology, Kharagpur, India

**Keywords:** Plant disease recognition, Deep learning, Transfer learning, Precision agriculture, Agroecology, Plant sciences, Computational models, Computational platforms and environments, Data acquisition, Image processing, Machine learning

## Abstract

Crop disease is a significant challenge in agriculture, requiring quick and precise detection to safeguard yields and reduce economic losses. Traditional diagnostic methods are slow, labor-intensive, and rely on expert knowledge, limiting scalability for large-scale operations. To overcome these challenges, a novel architecture called *Mob-Res*, combining residual learning with the *MobileNetV2* feature extractor, is introduced in this work. Despite having only 3.51 million parameters, *Mob-Res* is lightweight and well-suited for mobile applications while delivering exceptional performance. The proposed model is assessed using two benchmark datasets: *Plant Disease Expert*, consisting of 199,644 images across 58 classes, and *PlantVillage*, with 54,305 images across 38 classes. Through a rigorous training strategy, *Mob-Res* demonstrates robust performance, achieving 97.73% average accuracy on the *Plant Disease Expert* dataset and 99.47% on the *PlantVillage* dataset. The cross-domain validation rate (*CDVR*) is computed to assess its cross-domain adaptability, with the model showing competitive results compared to other pre-trained models. Additionally, *Mob-Res* outperforms prominent pre-trained *CNN* architectures, surpassing *ViT-L32* while maintaining a significantly lower parameter count and achieving faster inference times. The proposed model enhances interpretability by utilizing Gradient-weighted Class Activation Mapping (*Grad-CAM*), *Grad-CAM++*, and Local Interpretable Model-agnostic Explanations (*LIME*). These techniques provide visual insights into the neural regions influencing the predictions. The experimental results conducted in the current work highlight *Mob-Res* as a promising solution for automated plant disease detection, supporting large-scale agricultural operations and advancing global food security.

## Introduction

Crop diseases are a significant threat to agricultural yield, causing immense losses and affecting global food security^1,2^. These diseases spread through microorganisms like fungi, bacteria, viruses, and weather conditions such as temperature, humidity, and rainfall^3^. Therefore, diagnostic delays can lower yields, increase food insecurity, and raise prices. Hence, early detection is vital to prevent crop failures and economic losses^4–6^. Traditional visual inspections by experts are slow, labor-intensive, and error-prone, making them inefficient for large-scale farming^7^. To address these challenges, automated and efficient detection systems are increasingly necessary^8^. Recent advancements in Deep Learning (*DL*) have shown potential in improving the accuracy of plant disease detection and classification^9^.

Convolutional Neural Networks (*CNN*s) and transfer learning approaches have demonstrated superior performance compared to conventional machine learning techniques such as Support Vector Machines (*SVM*), Multi-Layer Perceptrons (*MLP*) and *k*-Nearest Neighbors (k-NN)^10^. However, despite their success, many state-of-the-art models are computationally expensive and require substantial processing power, limiting their applicability in real-world agricultural settings, particularly on resource-constrained devices^11^. Moreover, while vision transformers (*ViT*) and architectures such as Swin transformers^12^ have emerged as robust alternatives, their high computational demands make them impractical for deployment on edge devices used in agricultural environments^5^.

Another major limitation of existing deep learning-based approaches is their lack of interpretability in plant disease identification^9^. Most CNN-based models operate as black-box systems, making it difficult for farmers and agricultural experts to understand the decision-making process. This lack of transparency reduces trust in AI-based solutions and hinders widespread adoption in precision agriculture. To address these challenges, Explainable AI (*XAI*)^13^ techniques are increasingly being integrated into plant disease classification models to provide visual explanations of predictions, enhancing model transparency and interpretability.

In this study, we propose a novel hybrid deep learning model that combines *MobileNetV2* with residual blocks for efficient and interpretable plant disease classification. *MobileNetV2* ensures lightweight computation, making the model suitable for deployment on mobile and edge devices, while the residual blocks enhance feature extraction and classification accuracy. To improve model interpretability, we incorporate *XAI* techniques such as *Grad-CAM*, *Grad-CAM++*, and Local Interpretable Model-Agnostic Explanations (*LIME*). *Grad-CAM* and *Grad-CAM++* provide class-discriminative localization maps to highlight important regions influencing model decisions, whereas LIME offers a model-agnostic approach by perturbing input images and analyzing feature contributions. By leveraging these interpretability methods, we ensure that the model not only delivers high accuracy but also provides transparent decision-making insights, fostering trust among end users.

Furthermore, existing disease detection models trained on specific crops are often not directly adaptable due to variations in leaf structure, symptom presentation, and environmental factors^14^. Many prior studies rely on dataset-specific training, leading to poor generalization across different plant species^15^. Additionally, limited efforts have been made to integrate interpretability into these models, further restricting their practical utility^9^. Our approach addresses these gaps by developing a computationally efficient and explainable deep learning framework tailored for plant disease classification.

**Fig. 1:**
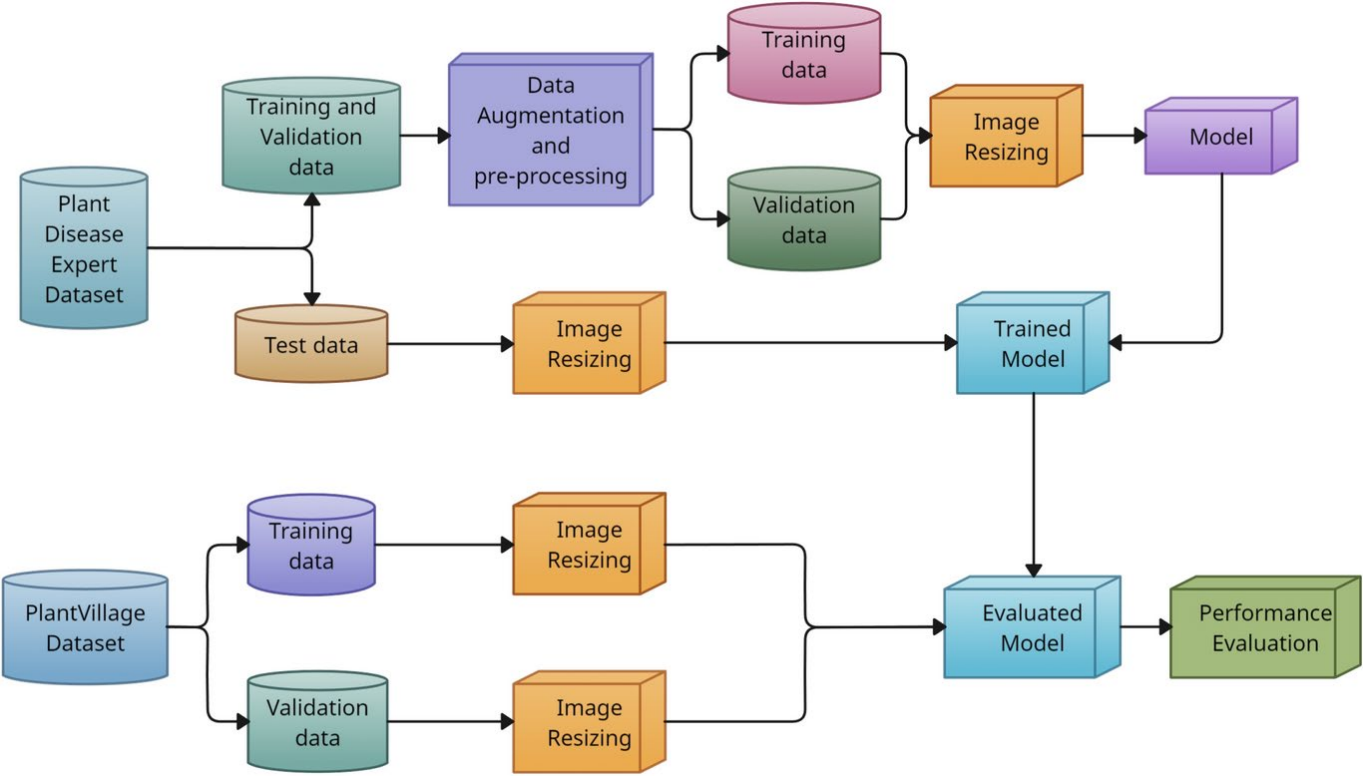
Proposed model work flowchart.

The overall workflow of the proposed approach is illustrated in Fig. 1. By combining computational efficiency, high classification accuracy, and enhanced interpretability, our model provides a scalable solution for real-time plant disease detection, ensuring early intervention and improved agricultural productivity.

## Related works

Agricultural researchers have turned to machine learning (*ML*), Deep Learning (*DL*), and image processing to revolutionize plant disease detection. A key breakthrough came in 2016, when researchers like Mohanty and colleagues, for example, used *GoogLeNet* with transfer learning to achieve a remarkable 99.34% accuracy in plant disease prediction^16^. Building on this, in 2017, Fuentes and his team explored models like *Faster R-CNN*, *R-FCN*, and *SSD* for classifying tomato diseases, showcasing the power of advanced deep learning models^17^.

Fast forward to 2019, when a team of researchers led by Geetharamani introduced a custom nine-layer *CNN* that outperformed widely used transfer learning methods, setting a new benchmark in disease detection accuracy^2^. Around the same time, Brahimi and colleagues proposed a teacher-student framework to improve the robustness of their models^9^. By 2020, attention shifted towards lightweight models, such as the shallow networks introduced by Li and his team, offering faster and more efficient predictions^18^.

In 2022, the study in^1^ improved teacher-student models, providing better generalization and faster training, while researchers in^12^ introduced the Convolutional Swin Transformer (*CST*), blending convolutional layers with transformer-based techniques for enhanced feature extraction. By 2023, the work in^19^ presented the *CCDL* architecture, which furter pushed the boundaries of crop disease classification. In 2024, the findings in^20^ achieved an impressive 99.89% accuracy with an ensemble model comprising *DenseNet201*, *EfficientNetB0*, and other cutting-edge architectures.

Although traditional deep learning models have delivered impressive results, researchers are increasingly developing hybrid *CNN* models for plant disease detection, combining powerful feature extraction capabilities of *CNNs* with various classification models^21^. In addition to these advancements, lightweight *CNN* models have demonstrated significant effectiveness in real-time applications. For example, a modified *AlexNet* with dilated convolution, *VGG-16* combined with Kernel Support Vector Machine (*SVM*), and a simple 6-layer *CNN* model have all achieved high *precision* in detecting crop diseases. As a result, researchers have shifted from traditional *ML* methods to pioneering architectures like transformers^12^ and parallel models^20^. Furthermore, cutting-edge techniques such as Explainable *AI* (*XAI*)^13^ are enhancing model transparency and making *DL* models more understandable. Consequently, integrating these innovative approaches has markedly improved the efficiency and reliability of modern models.

Despite these advancements, several research gaps remain:

- **Dataset-specific optimization:** Many previous works, such as those in^17^ and ^2^, demonstrated high accuracy but were trained and tested on a single dataset. This limits their ability to generalize across diverse datasets with different plant species, environmental conditions, and imaging variations.
- **Inconsistent interpretability:** While CNN models, such as those introduced in 2019^9^ and 2020^18^, improved accuracy, they lacked interpretability mechanisms, making it difficult for users to understand the decision-making process. Explainable AI (*XAI*) techniques remain underutilized in many works, restricting their practical adoption.
- **Lightweight design for field deployment:** Recent studies, such as ensemble model in^20^, prioritized accuracy but at the cost of computational efficiency. Many high-performing models are too resource-intensive for real-time field applications, limiting their practical use in agriculture.

To address these gaps, we propose *Mob-Res*, a parallel *CNN* model that balances accuracy, efficiency, and interpretability.

### Problem definition, motivation and contribution of the work

Recent studies highlight a shift from traditional methods like *SVM*, Multi-Layer Perceptron, and *k-NN* to deep learning techniques such as *CNN*s and transfer learning for plant disease classification. Although new architectures like *Swin Transformers* are being explored for their robustness, they face deployment challenges due to high computational demands. Additionally, the lack of interpretability in these cutting-edge models reduces trust and understanding, which is crucial for effective plant disease detection and treatment. Therefore, developing lightweight models that balance accuracy with computational efficiency and transparency is vital for practical agricultural applications.

Given the urgent need for efficient and interpretable models, this work focuses on plant disease classification. Current state-of-the-art models achieve impressive accuracy, but their high computational demands and lack of transparency limit their practical application. Our goal is to develop a lightweight and interpretable deep learning model. By combining *MobileNetV2* with residual blocks, we aim to create a model suitable for resource-constrained devices while ensuring transparent decision-making processes. This approach addresses existing limitations and makes advanced deep learning techniques more practical for agriculture.

Our paper advances plant disease classification by presenting a novel, efficient, and interpretable deep learning model that addresses key challenges in accuracy, computational efficiency, and decision transparency. The main contributions of this work are as follows:

**(1)** Combining the architecture of *MobileNetV2* with residual blocks, thereby making our model relatively lightweight compared to many state-of-the-art models, with only 3.51 M parameters. **(2)** Demonstrating the adaptability and generalization capability of the proposed model through experiments on two distinct datasets, utilizing cross-dataset validation and fine-tuning techniques. Comparing our proposed model to popular pre-trained models, we demonstrate its superior adaptability to varied data distributions, enhancing its effectiveness in real-world applications. **(3)** Achieving 99.45% accuracy and 99.43% *F1-Score* on *PlantVillage*, the proposed model surpasses recent state-of-the-art methods in plant disease identification.

**Fig. 2:**
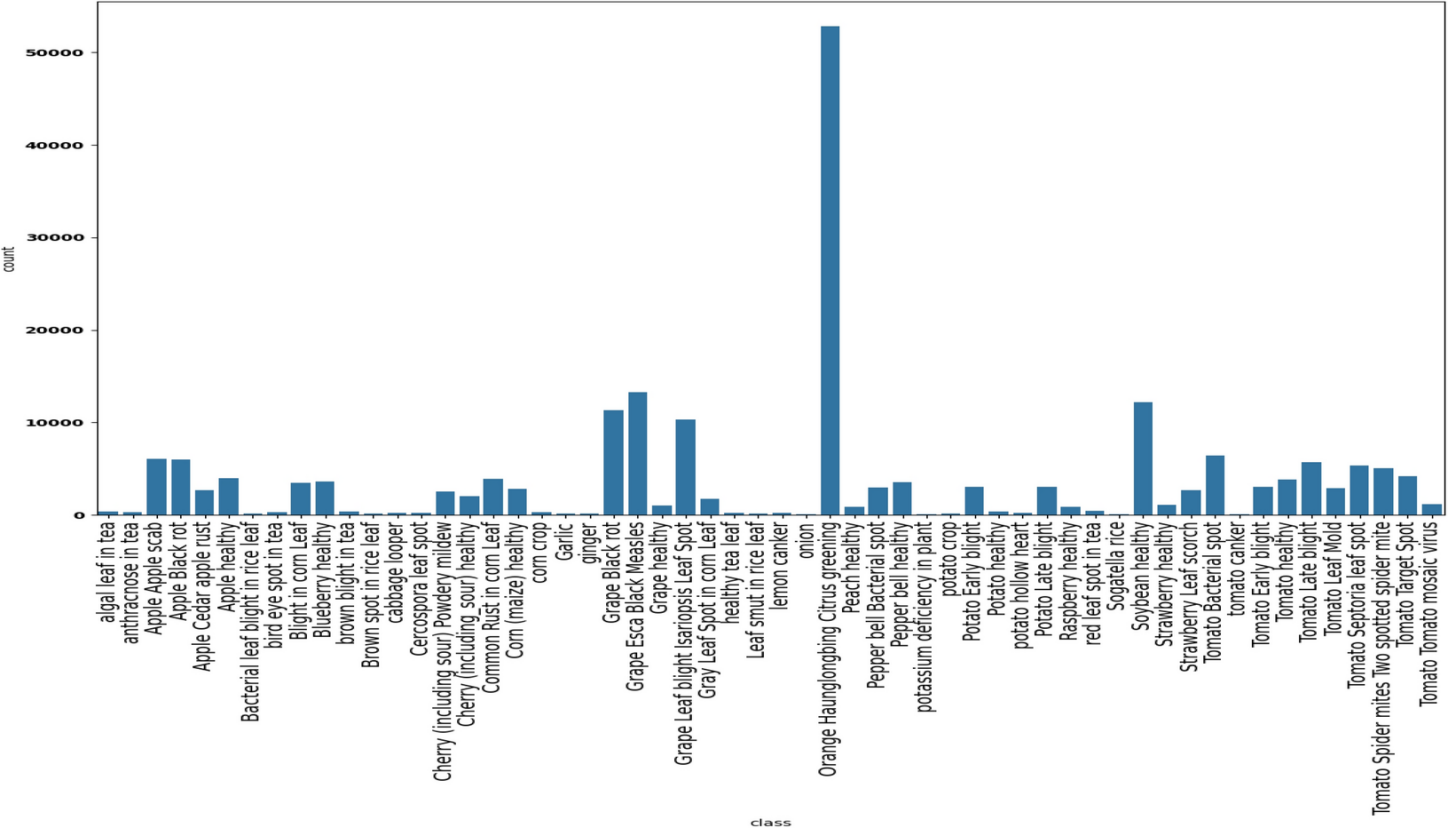
Classwise distribution of *Plant Disease Expert* dataset.

**(4)** Integrating Explainable AI (*XAI*) techniques like *Grad-CAM* enhances the interpretability of the model by revealing which neural regions influence its decisions. This transparency allows researchers to visualize and analyze the contributions of specific image regions to predictions, thereby improving trust in the model and aiding in performance refinement and validation.

## Materials and methods

The proposed model processes 128 $\times$ 128 $\times$ 3 input images normalized to [0, 1] to classify diseases. Fig. 1 presents the workflow of the proposed *Mob-Res* model. The datasets considered in the study are split into training, validation, and test sets as presented in Section 3.1. Section 3.2 discusses the architecture of the *Mob-Res*. We aim to provide a clear explanation of the methodology for easy replication.

### Dataset description

In our study, we utilize two publicly available datasets-*Plant Disease Expert* and *PlantVillage* to conduct the experiments for our research. A detailed description of each dataset is provided below.

**Plant disease expert**: The dataset used in our study, sourced from *Kaggle*^22^, includes 199,644 images across 58 classes. We focused on this dataset for model training but excluded the *Nitrogen deficiency in plant* and *Waterlogging in plant* classes due to their limited number of images. Despite this, the remaining 56 classes still show significant imbalance, as shown in Fig. 2. For instance, *Orange Haunglongbing Citrus greening* and *Grape Esca Black Measles* have 52,872 and 13,284 images, respectively, while *Potassium deficiency in plant* and *Tomato canker* have only 54 and 57 images. Most classes have between 300 and 5,000 images. To address this imbalance, we created a test dataset by selecting either 65 images or 10% of images per class, whichever was smaller, resulting in 2,662 test images and 196,948 training images. For classes with more than 1,100 training images, we have randomly sampled 1,100 images. For classes with fewer training images, we use augmentation techniques-such as flipping, rotations, and Gaussian noise-to boost their numbers to 1,100 images per class. This resulted in a balanced dataset with 56,000 training images and 5,600 validation images to ensure robust model performance across all classes.

**PlantVillage**: The *PlantVillage* dataset, a publicly available resource^23^, is widely used as bench-mark dataset in plant disease identification research. It includes 38 classes with 43,444 training images and 10,861 validation images. These *RGB* images, stored in *JPG* format, are captured in controlled laboratory environments, ensuring high-quality and standardized data. This extensive dataset supports the development of robust machine learning models for early disease detection and diagnosis, contributing to sustainable agricultural practices and improved food security. The dataset comprises 14 crop species: apple, blueberry, cherry, grape, orange, peach, pepper, potato, raspberry, soy, squash, strawberry, and tomato. It features images of 17 common diseases, four bacterial infections, two diseases caused by mold (oomycete), two viral diseases, and one disease caused by mites. Additionally, the dataset includes images of healthy leaves from 12 crop species that show no visible signs of disease.

### Proposed architecture

**Fig. 3:**
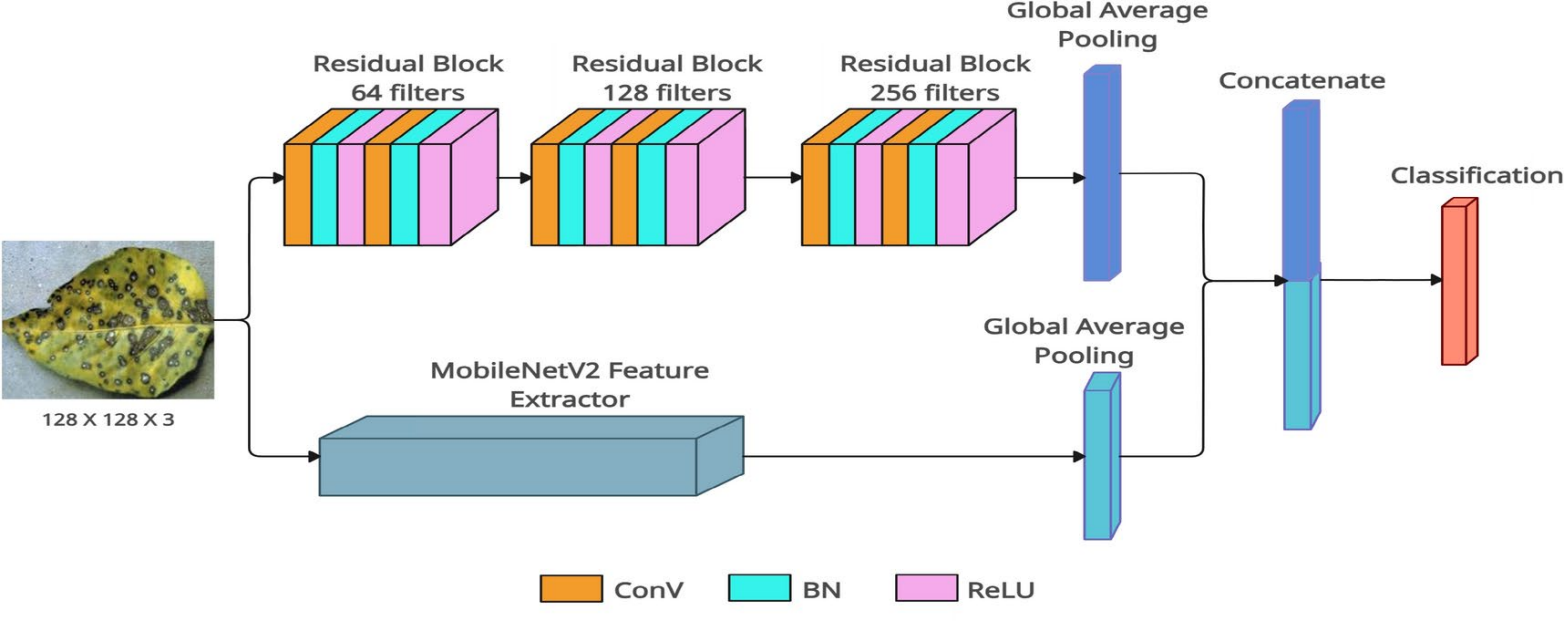
Block diagram of the proposed *Mob-Res* model for input image of 128 $\times$ 128 $\times$ 3. Here, *ConV* stands for Convolutional layer, *BN* stands for Batch Normalization layer.

The proposed model integrates two paths, *Path1* and *Path2*, to combine the strengths of residual-based learning and *MobileNetV2* for classifying 128 $\times$ 128 RGB images (see Fig. 3). *Path1* employs three residual blocks to enhance feature extraction, while *Path2* uses *MobileNetV2*, a lightweight network, as a feature extractor by omitting its top classification layer.

Our work lies in the synergistic integration of these two approaches to create a computationally efficient yet highly discriminative model for plant disease classification. *MobileNetV2* is chosen for its lightweight architecture and efficiency in mobile and embedded systems, making it ideal for real-time applications in resource-constrained environments. Unlike standard lightweight architectures, the proposed *Mob-Res* model strategically incorporates residual blocks to enhance feature propagation and gradient flow, mitigating the degradation problem often observed in deep CNNs. This improves learning efficiency without significantly increasing the parameter count, making it suitable for resource-constrained devices.

**Fig. 4:**
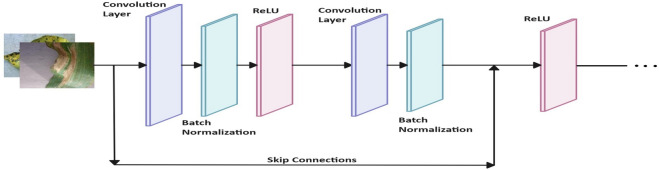
Residual block.

Let $\mathbf {\textit{X}}$ represent the input image with dimensions $H \times W \times C$. In *Path1*, residual blocks process the input through convolutional layers, batch normalization, and *ReLU* activation, with shortcut connections to adjust dimensions as needed, as shown in Fig. 4. The features from both paths are then concatenated to produce the final feature representation, as Eqn. (1):

1 $$\begin{aligned} \mathbf {{F}}_{{final}} = \mathcal {F}\left( \textbf{F}_{{Mobile}}, \textbf{F}_{{Res3}}\right) , \end{aligned}$$

where, ${\mathcal {F}}(\cdot )$ denotes the fusion operation.

*Path1* consists of stacked residual blocks with increasing filter numbers, ending in global average pooling to produce a feature vector. *Path2* uses *MobileNetV2*, pre-trained on *ImageNet* and initialized without its top layer, followed by global average pooling. The feature vectors from both paths are then concatenated, combining their strengths. A fully connected dense layer with *softmax* activation generates class probabilities. The following sections will provide further details on the model components.

### Path1: Residual path

Path1 consists of three residual blocks designed to progressively increase the number of filters and enhance feature extraction capabilities. The input image, with dimensions $128 \times 128 \times 3$, first passes through a residual block with 64 filters, where the input is processed by a $3 \times 3$ convolutional layer followed by batch normalization and *ReLU* activation. A second convolutional layer with 64 filters is applied, followed by batch normalization, and a skip connection is introduced to align the input and output dimensions using a $1 \times 1$ convolution. The output of this block has the dimensions $128 \times 128 \times 64$. The second residual block processes the output of the first block through a convolutional layer with 128 filters, batch normalization, and *ReLU* activation, followed by a second convolutional layer with 128 filters, batch normalization, and another skip connection. The output dimensions of this block are $128 \times 128 \times 128$. The third block further processes the output of the second block with a convolutional layer with 256 filters, followed by batch normalization and ReLU activation, and a second convolutional layer with 256 filters, batch normalization, and a skip connection to align dimensions. The output of this block has the dimensions $128 \times 128 \times 256$. At the end of the third block, a Global Average Pooling layer is applied to reduce the spatial dimensions, resulting in a feature vector of size $1 \times 256$, which encapsulates the features extracted from Path1. Path1 thus consists of three residual blocks, each with growing filter counts. The outputs of these residual blocks are detailed in Eqn. (2)-(4):

2 $$\begin{aligned} \mathbf {\textit{F}}_{{{Res1}}} =&\mathscr {R}_1(\mathbf {\textit{X}}), \end{aligned}$$

3 $$\begin{aligned} \mathbf {\textit{F}}_{{{Res2}}} =&\mathscr {R}_2(\mathbf {\textit{F}}_{{{Res1}}}), \end{aligned}$$

4 $$\begin{aligned} \mathbf {\textit{F}}_{{{Res3}}} =&\mathscr {R}_3(\mathbf {\textit{F}}_{{{Res2}}}), \end{aligned}$$

where, $$\quad \mathscr {R}_1: \mathbb {R}^{H \times W \times C} \rightarrow \mathbb {R}^{H_1 \times W_1 \times 64}, \quad \mathscr {R}_2: \mathbb {R}^{H_1 \times W_1 \times 64} \rightarrow \mathbb {R}^{H_2 \times W_2 \times 128}, \quad \mathscr {R}_3: \mathbb {R}^{H_2 \times W_2 \times 128} \rightarrow $$$$\mathbb {R}^{H_3 \times W_3 \times 256}.$$ In this context, $$\mathbf {\textit{F}}_{{{Res1}}},~\mathbf {\textit{F}}_{{{Res2}}},~and~\mathbf {\textit{F}}_{{{Res3}}}$$ represent the feature maps after each residual block, and $$H_i$$, $$W_i$$ denote the height and width of the feature maps of the *i*th residual block.

### Path2: *MobileNetV2*

*MobileNetV2*, a lightweight deep neural network architecture, is employed as the backbone of the second path. It is pre-trained on the *ImageNet* dataset, providing a robust feature extractor for the input images. *MobileNetV2* is initialized without its top classification layer, allowing us to use it purely as a feature extractor. The output feature maps of *MobileNetV2* have a dimension of 4 $\times$ 4 $\times$ 1280 and are globally averaged to produce a compact feature vector of size 1 $\times$ 1280, as is done in *Path1*.

The second path, $\mathscr {M}(\mathbf {\textit{X}})$, utilizes *MobileNetV2* as the feature extractor with the output being represented by Eqn. (5):

5 $$\begin{aligned} \mathbf {\textit{F}}_{{{Mobile}}} = \mathscr {M}(\mathbf {\textit{X}}), \end{aligned}$$

where, $\textit{F}_{{{Mobile}}}$ represents the feature map extracted by *MobileNetV2*, feature vectors of dimensions $1 \times 256$ from *Path1* and $1 \times 1280$ from *Path2* are concatenated to form a $1 \times 1536$ vector. This combined feature vector is then processed through a fully connected dense layer with a *softmax* activation function, which outputs the final class probabilities. The prediction, $\hat{y}$, is obtained by feeding $\mathbf {\textit{F}}_{final}$ into a classifier, followed by the *softmax* function, as described in Eqn. (6):

6 $$\begin{aligned} \hat{y} = \text {softmax}\left( \mathbf {\textit{W}} \cdot \mathbf {\textit{F}}_{{{final}}} + \mathbf {\textit{b}}\right) . \end{aligned}$$

Here, $\mathbf {\textit{W}}$ represents the weight matrix of the classifier, while $\mathbf {\textit{b}}$ denotes the bias vector. The code of the architecture is available at https://github.com/Chiranjit369/Mob-Res.

### Explainable AI

Explainable AI (*XAI*) is critical for enhancing the transparency and interpretability of deep learning models, especially in applications such as plant disease detection, where understanding the model’s decision-making process is crucial. In this study, we leverage three powerful *XAI* techniques: *Grad-CAM*^13^, *Grad-CAM++*^24^, and *LIME*^13^ to provide a comprehensive interpretation of the decision-making process of the proposed Mob-Res model. *Grad-CAM* works by computing the gradient of the class score with respect to the feature maps of the last convolutional layer, then using this gradient to weight the feature maps. The resulting weighted feature maps are combined to produce a heatmap that highlights the regions in the image most relevant to the model’s prediction. While *Grad-CAM* provides valuable insights into which regions influence the prediction, it can sometimes struggle with precise localization, particularly when multiple discriminative features exist within the image. To address this limitation, we incorporated *Grad-CAM++*, an extension of *Grad-CAM* that improves localization by incorporating higher-order gradients. This method captures more detailed information, particularly when multiple objects or features are involved, providing a more accurate localization of the discriminative regions. Additionally, we utilize *LIME* (Local Interpretable Model-agnostic Explanations), a model-agnostic approach that explains the predictions of black-box models by approximating the model locally around the prediction. *LIME* works by perturbing the input data, creating multiple variations of the image, and observing how these changes affect the model’s output. It then trains a locally interpretable surrogate model to identify which parts of the image (typically represented as superpixels) are most important for the model’s decision. The use of *LIME* enables us to identify the most critical areas that drive the predictions in a transparent manner. Together, *Grad-CAM*, *Grad-CAM++*, and *LIME* improve the transparency of the Mob-Res model, providing insights into its decision-making process and helping to validate its predictions, thereby ensuring its reliability in plant disease detection tasks.

## Results and discussions

This section evaluates the proposed model and discusses its experimental setup, performance, evaluation metrics, training strategies and hyperparameters. It benchmarks the model against top deep learning models, examines its parallel architecture, compares it with state-of-the-art models, and uses *Grad-CAM* to assess interpretability.

The deep learning models are trained and evaluated on a high-performance computer with notable specifications, including an Intel Core i5 12400 processor, 16 GB of DDR4 RAM, and a single NVIDIA RTX 3060 Ti graphics card. The graphics card features 8 GB of GDDR6 memory, 4,864 CUDA cores, and 152 tensor cores. All experiments on the pre-trained models are conducted using the latest stable version of PyTorch framework, while experiments on the *Mob-Res* model are performed using TensorFlow 3.10 with NVIDIA CUDA support.

### Performance metrics used

The evaluation of all considered models, including our proposed model, includes *accuracy*^19^, *precision* (Positive Predictive Value)^20^ and *recall* (True Positive Rate)^9^. These metrics comprehensively assess model performance on the *Plant Village* dataset. We have also introduced Cross-Domain Validation Rate (*CDVR*) to explain the cross-domain feature identification. It measures how well a model trained on one dataset (source domain) can predict accurately on another dataset (target domain) with differing distributions. High *CDVR* indicates the generalization and adaptability of the model to variations in data collection images and class distributions. Mathematically, it is expressed as the accuracy of a model trained on a source domain $D_s$ and evaluated on a target domain $D_t$, and the formula is as defined in Eqn. (7):

7 $$\begin{aligned} {CDVR}^{ D_s\rightarrow D_t}_{\text {cross}} = \frac{1}{N} \sum _{i=1}^{N} \mathbb {g}( \hat{y}_i = y_i), \end{aligned}$$

where, $N$ is the number of samples in the target domain $D_t$, $\hat{y}_i$ is the predicted label for sample $i$, $y_i$ is the true label for sample $i$, and $\mathbb {g}(\cdot )$ is the indicator function, which equals 1 if the prediction is correct $(\hat{y}_i = y_i)$ and 0 otherwise.

### Training strategies

In this study, various training strategies are applied to improve plant disease classification using the *Plant Disease Expert* and *PlantVillage*. Data augmentation enhances generalization, while transfer learning aids faster convergence, especially with limited labeled data. The proposed model uses an input resolution of 128$\times$128$\times$3 with a batch size of 16, whereas others use 224$\times$224$\times$3 with the same batch size. Key hyperparameters include the *Adam* optimizer with default momentum ($\beta _1 = 0.9$, $\beta _2 = 0.9$) and a 0.001 learning rate, and 40 epochs with early stopping to restore the best weights. *Grad-CAM*, *LIME* and *Grad-CAM++* are applied to visualize critical regions at the *ReLU* activation in layer 27 of *Path1* and the *out_relu* activation in layer 153 of *Path2*. This helps interpret how each pathway processes information. Intra-dataset and cross-dataset evaluations further assess model performance and adaptability across both datasets. All datasets are validated by domain experts from Indian Council of Agricultural Research (*ICAR*), ensuring reliable data quality for training and testing.

**Table 1:** Performance metrics of models on *Plant Disease Expert* (*A*) and *PlantVillage* (*B*) for different training strategies along with the average inference time (*IT*): A (Trained/validated on A), BA_f_ (Trained on B, fine-tuned on A), (A+B)A_f_ (Trained on combined A and B, fine-tuned on A), B (Trained/validated on B), AB_f_ (Trained on A, fine-tuned on B), (A+B)B_f_ (Trained on combined A and B, fine-tuned on B), AB (Trained on A, validated on B), BA (Trained on B, validated on A). Configurations in bolditalic columns signify generalization capability, while the rest of the configurations denote intra-domain adaptability of the model.

Name of the Model	A	BA_f_	(A+B)A_f_	A_avg._	B	AB_f_	(A+B)B_f_	B_avg._	*AB*	*BA*	*CDVR*_*avg.*_	IT (ms)
ResNet50^25^	95.76	88.63	96.12	93.50	97.10	98.95	98.32	98.12	89.84	91.03	90.44	4.11
DenseNet201^25^	97.53	93.37	95.76	95.55	97.47	99.04	98.95	98.49	94.53	91.81	93.17	4.94
ConvNeXt-base^26^	95.52	92.37	95.76	94.55	94.67	97.03	97.34	96.35	85.30	84.18	84.74	15.38
ShuffleNetV2 x2.0^27^	94.77	87.77	95.16	92.57	97.10	98.16	98.37	97.88	94.44	90.34	92.39	0.59
SqueezeNet 1.0^27^	**98.08**	86.48	**98.38**	94.31	95.80	99.27	98.76	97.94	85.47	84.43	84.95	0.82
MobileNetV2^25^	96.43	92.23	97.23	95.30	96.79	97.23	98.12	97.38	93.75	87.34	90.55	**0.25**
MobileNetV3Large^28^	96.39	92.37	96.17	94.98	95.93	97.45	99.01	97.46	92.39	86.14	89.27	0.27
ResNeXt50 32x4d^26^	95.98	91.63	96.42	94.68	96.30	98.32	98.01	97.54	89.22	90.44	89.83	4.26
GoogLeNet^21^	95.08	84.96	96.35	92.13	95.99	96.31	97.88	96.73	88.06	89.61	88.84	1.50
ViT-L32^29^	97.37	95.03	97.63	96.68	97.57	99.17	99.05	98.60	95.02	90.37	92.70	15.27
ViT-B32^29^	96.92	93.97	97.03	95.97	97.80	98.93	99.31	98.68	94.49	**92.97**	93.73	4.37
Swin V2B^12^	97.15	90.89	97.39	95.14	97.85	99.13	99.79	98.92	84.99	89.84	87.42	17.08
Swin V2S^12^	96.54	90.12	96.94	94.53	97.44	98.97	99.10	98.50	85.30	89.06	87.18	9.72
**Mob-Res**	97.52	**97.71**	97.97	**97.73**	**99.03**	**99.45**	**99.92**	**99.47**	**97.64**	92.67	**95.16**	5.98

### Performance comparison with pre-trained models

In this section, we analyze the performance of several widely used state-of-the-art deep learning models for plant disease classification, including *ResNet50*^25^, *DenseNet201*^25^, *ConvNeXt-base*^26^, *ShuffleNetV2 x2.0*^27^, *SqueezeNet 1.0*^27^, *MobileNetV2*^25^, *ResNeXt50 32x4d*^26^, *GoogLeNet*^21^, *ViT-L32*^29^, *ViT-B32*^29^, *Swin V2B*^12^ and *Swin V2S*^12^, and compare them against our proposed framework. Our goal is to evaluate both generalization and domain adaptability using the *Plant Disease Expert* and *PlantVillage* datasets. This analysis will reveal strengths and weaknesses of each model, guiding their applicability in practical plant disease classification tasks.

The *Mob-Res* model demonstrates strong generalization and domain adaptation capabilities through its performance across various cross-domain and intra-domain configurations as highlighted in Table 1. *Mob-Res* achieves a remarkable *CDVR* of 97.64% in the *AB* configuration, indicating a successful transfer of knowledge from the more complex Dataset *A* to the simpler Dataset *B*. However, the *CDVR* decreases to 92.67% in the *BA* configuration, reflecting the challenges of generalizing from datasets with fewer to more diverse classes. Notably, *Mob-Res* maintains a *CDVR* exceeding 92% in both scenarios, outperforming models such as *Swin V2B* and *SqueezeNet 1.0* with lower *CDVR*.

**Fig. 5:**
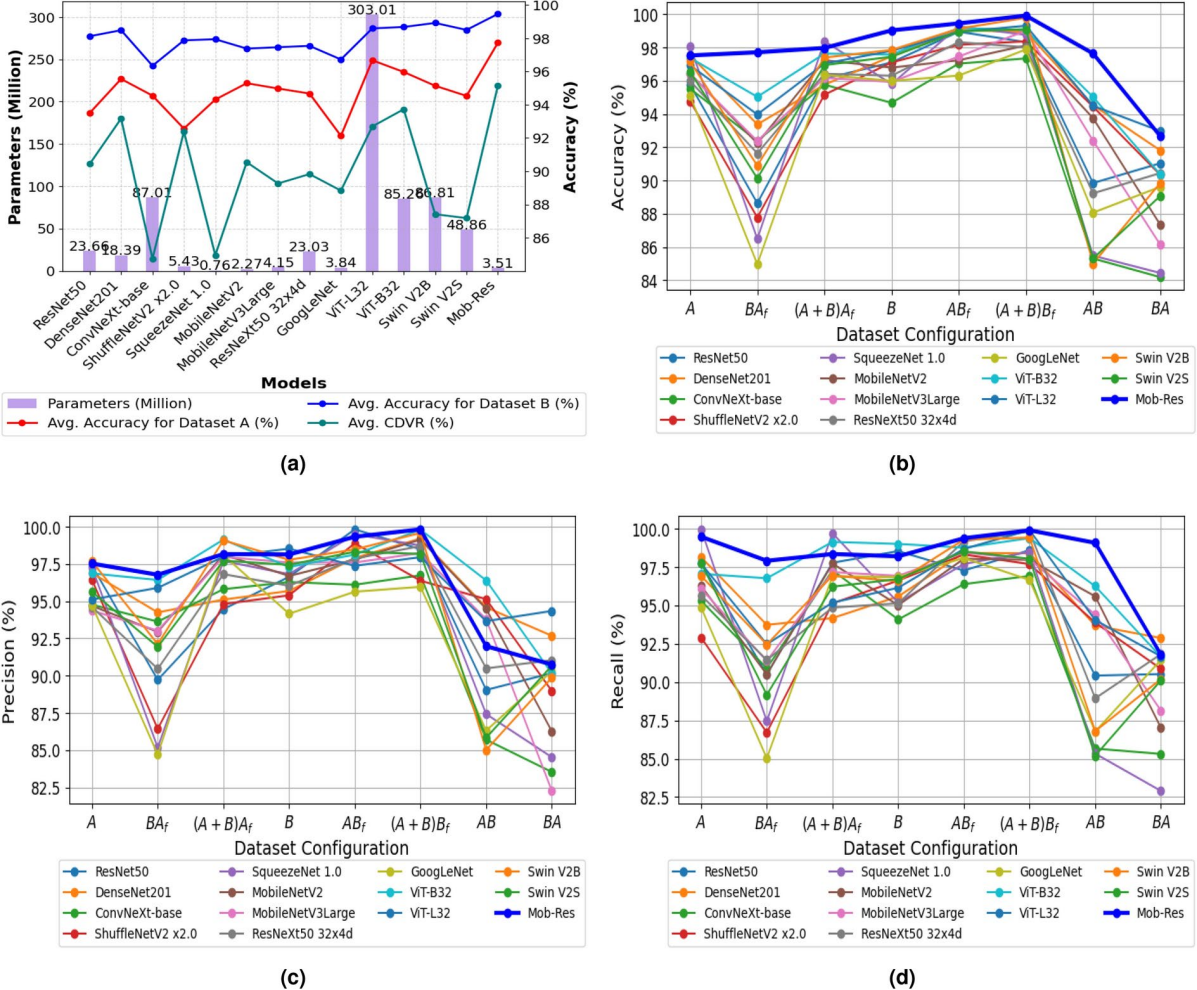
Model analysis across different dataset configurations: (**a**) Analysis of parameters in millions (M), along with the average accuracies on Datasets A and B and the average *CDVR* for various models; (**b**) Accuracy, (**c**) Precision and (**d**) Recall trends for each model across different training configurations illustrated using line plots emphasize performance variations and consistencies.

In terms of intra-domain capabilities, *Mob-Res* performs well with an accuracy of 97.52% on *A* (trained and validated on *A*) configuration and *99.03%* on *B* (trained and validated on *B*) configuration as seen in Fig. 5b, highlighting strong feature extraction from given datasets. In terms of fine-tuning capabilities, *Mob-Res* achieves 99.45% in the ${AB_{f}}$ configuration (trained on *A*, fine-tuned on *B*), outperforming *SqueezeNet 1.0* and *ViT-L32*. In the combined fine-tuning scenario ${(A+B)B_{f}}$, it reaches 99.92%, surpassing *Swin V2B* and *ViT-B32*. These results underscore the superior adaptability of the *Mob-Res* in transitioning between datasets of varying complexities. Although *SqueezeNet 1.0* shows slightly better accuracy in specific intra-domain settings (98.08% on *A*), *Mob-Res* consistently balances intra-domain performance and cross-domain adaptability, achieving notable gains in fine-tuning scenarios.

In addition to accuracy, the models are evaluated using *precision* and *recall* to offer a more complete assessment of their ability to distinguish between classes. Specifically, Fig. 5c illustrates model performance in terms of *precision*, while Fig. 5d compares the models based on *recall* across various dataset configurations. These evaluations highlight the discriminative capabilities of *Mob-Res*, particularly in challenging scenarios.

To highlight the efficiency of *Mob-Res*, Fig. 5a showcases its performance relative to the parameter count, emphasizing its lightweight design. *Mob-Res* scores the highest average accuracy on both datasets A and B and the highest *CDVR*. *Mob-Res* effortlessly outperforms heavy models like *ViT-L32* (303.01 M), *ViT-B32* (85.26 M), and *DenseNet201* (18.39 M) with a mere 3.51 M parameters. Although *SqueezeNet 1.0* (0.76 M) is the lightest, it exhibits notable performance variability (see Table 1).

**Table 2:** Comparison of individual components with the complete architecture across *Plant Disease Expert* and *PlantVillage*.

	*Plant Disease Expert*			*PlantVillage*		
Name of the Model	Accuracy	Precision	Recall	Accuracy	Precision	Recall
Residual Blocks	93.20	90.98	92.73	95.29	95.12	93.97
MobileNetV2	96.43	96.96	98.53	93.75	90.39	94.82
**Mob-Res**	**97.52**	**97.53**	**98.02**	**99.45**	**99.33**	**99.37**

Based on the experiments, *Mob-Res* demonstrates strong performance across different dataset configurations, achieving high accuracy in various scenarios. *Mob-Res* demonstrates strong generalization capabilities, achieving performance comparable to more advanced models even with a lightweight design. Overall, *Mob-Res* offers a balanced approach to robustness, efficiency, and generalization, making it highly effective for plant disease classification.

We now present the ablation study in Table 2, which unveils the critical role of each component within the *Mob-Res* architecture. By comparing three configurations: residual blocks only, *MobileNetV2* only, and the combined *Mob-Res* model-this study offers a compelling insight into how each element shapes the overall performance. The residual blocks achieve an accuracy of 93.20% on the *Plant Disease Expert* dataset and 95.29% on *PlantVillage*, while *MobileNetV2* obtains an accuracy of 96.43% on *Plant Disease Expert* and 93.75% on *PlantVillage*. The combined *Mob-Res* model enhances the accuracy, reaching 97.52% on *Plant Disease Expert* and 99.45% on *PlantVillage*.

**Fig. 6:**
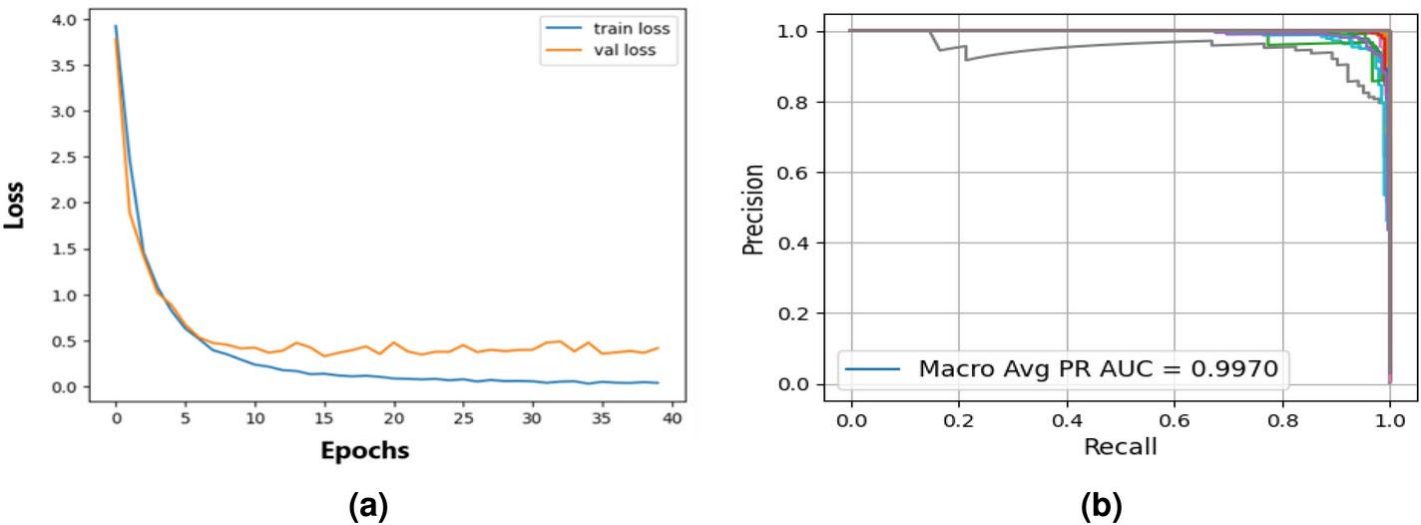
Model performance evaluation: (**a**) Loss Curve of *Mob-Res* on *PlantVillage*, (**b**) *P-R* curve of *Mob-Res* on the *PlantVillage*.

Fig. 6a shows the loss curve for Mob-Res. The loss curves reveal that Mob-Res consistently maintains lower loss values and converges more smoothly throughout the training process, suggesting reliable learning and stable training performance. Fig. 6b shows a Precision-Recall (*P-R*) curve for a classification model, where precision (y-axis) and recall (x-axis) are evaluated across different thresholds. The macro-average *P-R* area under curve (*AUC*) of 0.9970 indicates the model has near-perfect performance, balancing high precision and recall across all classes. This suggests the model is highly effective at correctly identifying positive instances with minimal misclassification.

**Fig. 7:**
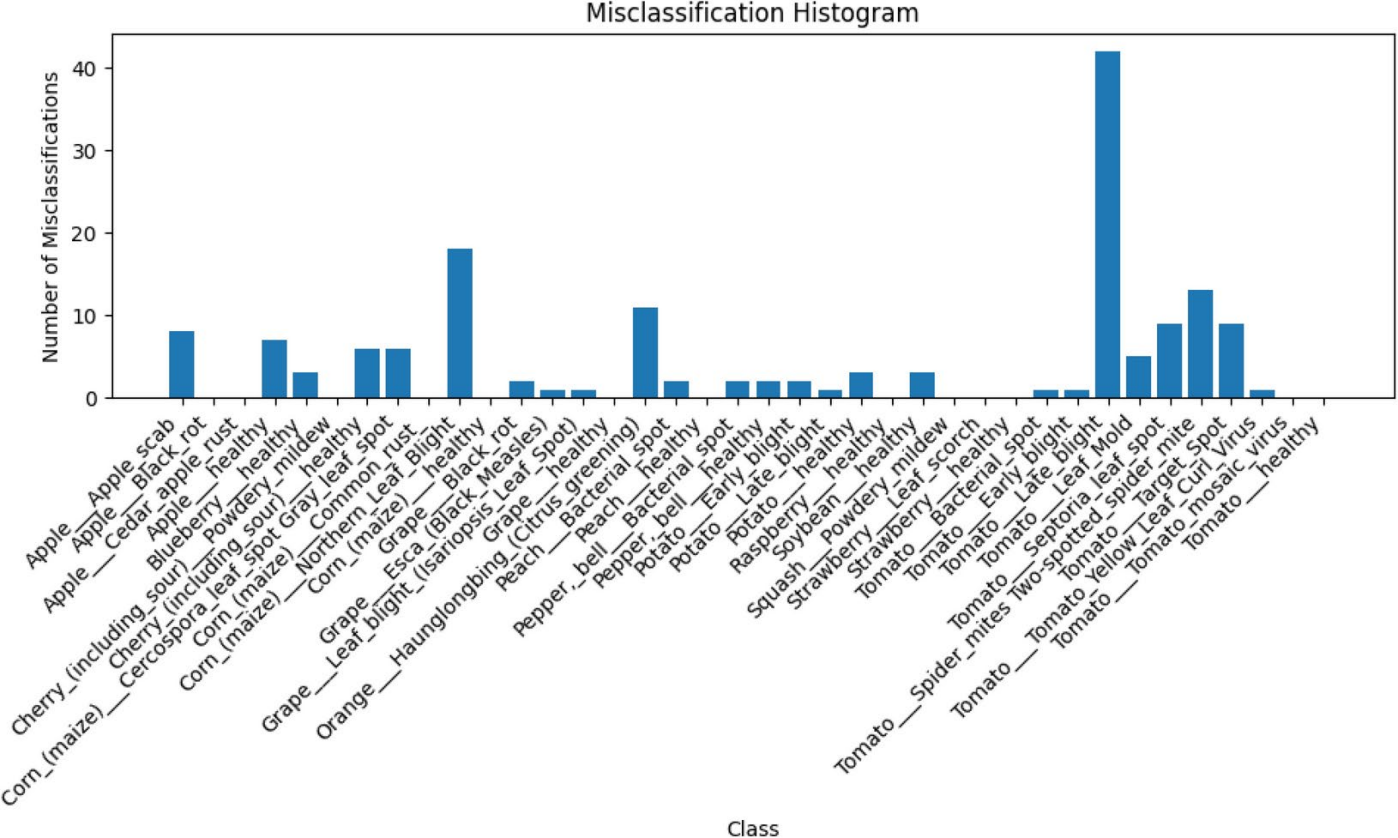
Misclassification distribution generated by *Mob-Res* highlighting misclassification counts on *PlantVillage*.

The misclassification bar graph in Fig. 7 reveals several insights into the model’s performance across various plant disease categories. The class *Tomato Late blight* exhibits the highest number of misclassifications at *42*, which suggests significant difficulty in distinguishing this disease from others, likely due to visual similarities with related conditions. Additionally, the model misclassified *13* instances of *Tomato Septoria leaf spot* and *9* instances of *Tomato Target Spot*, indicating a challenge in accurately classifying diseases within the tomato category. Conversely, certain classes, such as *Apple Black rot*, *Corn Common rust*, and *Grape Esca (Black Measles)*, show *0* misclassifications, demonstrating the model’s robustness in identifying these diseases. Among the grape-related diseases, *Grape Black rot* had *2* misclassifications, while *Grape Leaf blight* had only *1* misclassification, reflecting relatively strong performance. Healthy crops like *Apple*, *Blueberry*, and *Strawberry* also exhibit *0* misclassifications, showcasing the model’s ability to accurately identify healthy samples. However, the *18* misclassifications for *Corn Northern Leaf Blight* and *11* for *Orange Haunglongbing (Citrus greening)* highlight areas where the model struggled to differentiate specific diseases with more distinct characteristics. Overall, while the model performs well for many disease categories, the misclassification rates for tomato-related diseases and certain corn and citrus diseases suggest that additional refinements are necessary, especially in handling visually similar symptoms.

The *Mob-Res* model demonstrates excellent effectiveness in plant disease classification, balancing high accuracy with computational efficiency, making it suitable for deployment on resource-constrained devices. With a lightweight architecture combining *MobileNetV2* and residual connections, it achieves competitive performance, including a classification accuracy of 97.73% on dataset A and 99.47% on dataset B as shown in Table 1, while maintaining low computational overhead. The model’s ability to process data quickly, with an average inference time of 5.98 ms, highlights its efficiency on resource-constrained devices for real-time applications. The misclassification analysis in Fig. 7 highlights the strong classification performance of the proposed model, showing zero misclassifications in several classes, such as Apple Cedar Apple Rust, Cherry Powdery Mildew, Corn Common Rust, and Tomato Mosaic Virus. However, the model struggles most with Tomato Late Blight, recording 42 misclassifications, followed by Tomato Septoria Leaf Spot with 13 misclassifications, among others. Despite these challenges, low loss values in Fig. 6a and the precision-recall curve in Fig. 6b indicate overall robust performance. However, further refinement is needed to improve the model’s ability to differentiate between these similar crops and disease types. This will ensure better generalization for real-world agricultural applications. To demonstrate the robustness of the proposed model, we have also evaluated it on a sugarcane field data set, further validating its effectiveness on the field data set.

### Experiments on field dataset

We fine-tuned our *Mob-Res* model on the Sugarcane Leaf Disease Dataset^30^, which contains 2,569 sugarcane leaf images across five classes. The dataset was randomly split into 90:10 proportions, resulting in 2,269 training images and 250 testing images. The models listed in Table 1, including our *Mob-Res* model, are evaluated on this dataset. While the other models were initialized with pretrained *ImageNet* weights, only our *Mob-Res* model is tested both with and without fine-tuning. For the pretrained models, finetuning is performed by training only the final layer, and for finetuning *Mob-Res*, the model trained on a combined dataset of *Plant Disease Expert* and *PlantVillage* ((A+B) configuration described in Table 1) is taken and the final layer is retrained. Hyperparameters like batch size, number of epochs and others are kept consistent with the experiments in Table 1. The performance results are summarized in Table 3.

**Table 3:** Performance metrics of the models on Sugarcane dataset.

Model	Accuracy	Precision	Recall	F1-Score	Model	Accuracy	Precision	Recall	F1-Score
ResNet50^25^	84.00	84.67	83.76	83.74	GoogLeNet^21^	82.00	81.94	82.06	81.95
DenseNet201^25^	89.80	89.91	89.73	89.82	ViT-L32^29^	87.60	87.54	87.59	87.50
ConvNeXt-base^26^	**90.60**	89.67	**90.35**	89.78	ViT-B32^29^	88.80	88.90	88.71	88.67
ShuffleNetV2 x2.0^27^	89.60	90.80	89.47	**90.13**	Swin V2B^12^	88.40	88.48	88.32	88.35
SqueezeNet 1.0^27^	86.00	86.28	85.91	85.88	Swin V2S^12^	87.60	87.92	87.61	87.66
MobileNetV2^25^	87.70	88.92	87.88	88.12	Mob-Res (no finetune)	88.00	**89.96**	87.87	87.90
MobileNetV3Large^25^	87.68	88.85	86.51	87.76	Mob-Res (finetune)	85.60	85.76	85.50	85.53
ResNeXt50 32x4d^26^	86.40	86.33	86.34	86.30					

The proposed *Mob-Res* model demonstrates strong performance across multiple datasets, including an external sugarcane field dataset, reinforcing its robustness in real-world agricultural scenarios. *Mob-Res* achieves the highest precision (89.96%) among the models considered, indicating its effectiveness in minimizing false positives. On the benchmark dataset, *Mob-Res* achieves an *F1-Score* of 87.90%, outperforming *MobileNetV3Large* (87.76%) and *SqueezeNet* (85.88%), while maintaining a lightweight architecture suitable for deployment on resource-constrained devices. Furthermore, while *DenseNet201* (89.82%) and *ConvNeXt-base* (89.78%) achieve slightly higher* F1-Score*s, they come at the cost of significantly higher parameter counts and computational demands. In contrast, *Mob-Res* strikes an optimal balance between accuracy, computational efficiency, and interpretability. Additionally, when fine-tuned on an external sugarcane dataset, *Mob-Res* achieves an *F1-Score* of 85.53%, further validating its adaptability to different datasets and its capability to generalize beyond controlled benchmark datasets.

### Comparison with recent state-of-the-art works

This section compares our proposed *Mob-Res* model with recent state-of-the-art works, as compiled in Table 4. The listed architectures were trained on *PlantVillage*, and their validation accuracies are provided. To align with this configuration, we validated *Mob-Res* on *PlantVillage* after three training strategies: training on *PlantVillage* alone, training on *Plant Disease Expert* and fine-tuning on *PlantVillage*, and training on the combined *Plant Disease Expert* and *PlantVillage* followed by fine-tuning on *PlantVillage*. These approaches yielded accuracies of 99.03%, 99.45%, and 99.92%, respectively. For comparison purposes, we calculated the average accuracy across the three scenarios, resulting in 99.47%.

**Table 4:** Comparison with recent state-of-the-art works on *PlantVillage*.

Models	Param Count	Dataset	Accuracy
GhostNet^3^	2.60 M	PlantVillage	96.18
CNN using Attention^8^	0.70 M	PlantVillage	92.79
Teacher/Student^9^	67.00 M	PlantVillage	98.10
CNN^4^	27.00 M	PlantVillage	91.25
INC-VGGN^7^	-	PlantVillage	96.82
VGG-ICNN^3^	6.00 M	PlantVillage	99.16
9-layer deep CNN^2^	-	PlantVillage	96.46
PCCDL-PSCT^19^	-	PlantVillage	98.14
**Mob-Res**	**3.51 M**	**PlantVillage**	**99.47**

The summarization in Table 4 reveals that the *Mob-Res* model achieves an impressive 99.47% accuracy on *PlantVillage*, using just 3.51 M parameters. Despite its moderate complexity, *Mob-Res* surpasses other lightweight models like *GhostNet*, which has 2.60 M parameters and achieves 96.18% accuracy, and the *CNN* with attention from^8^, which achieves 92.79% accuracy with 0.70 M parameters. Even larger models, such as the Teacher/Student network (98.10% accuracy) with 67.00 M parameters^9^ do not match the performance of *Mob-Res*. This results demonstrate the capability of *Mob-Res* to deliver top-tier performance with significantly fewer computational resources. *Mob-Res* shows a greater average accuracy of 99.47% compared to 99.16% of *VGG-ICNN* with 6.00 M parameters. Despite having more parameters, many of the listed models (*VGG-ICNN*, *CNN*, *Teacher/Student*) fall short in accuracy compared to *Mob-Res*. Traditional *CNNs*, such as the one studied in^4^ with 27.00 M parameters and an accuracy of 91.25%, are also outperformed by *Mob-Res*. This highlights the efficiency of *Mob-Res*, achieving higher accuracy with significantly fewer parameters. Additionally, models like *INC-VGGN*^7^ with 96.82% accuracy and *PCCDL-PSCT*^19^ with 98.14% accuracy are all surpassed by *Mob-Res*, making it the preferred choice for applications that require both high precision and computational efficiency. The results demonstrate *Mob-Res* model outperforming many current methods in plant disease classification. Its lightweight nature makes it ideal for mobile platforms and leaf disease identification. In summary, *Mob-Res* achieves a notable balance between parameter count and accuracy, offering a highly effective solution with superior performance and efficiency across diverse classes in the *PlantVillage* dataset.

**Fig. 8:**
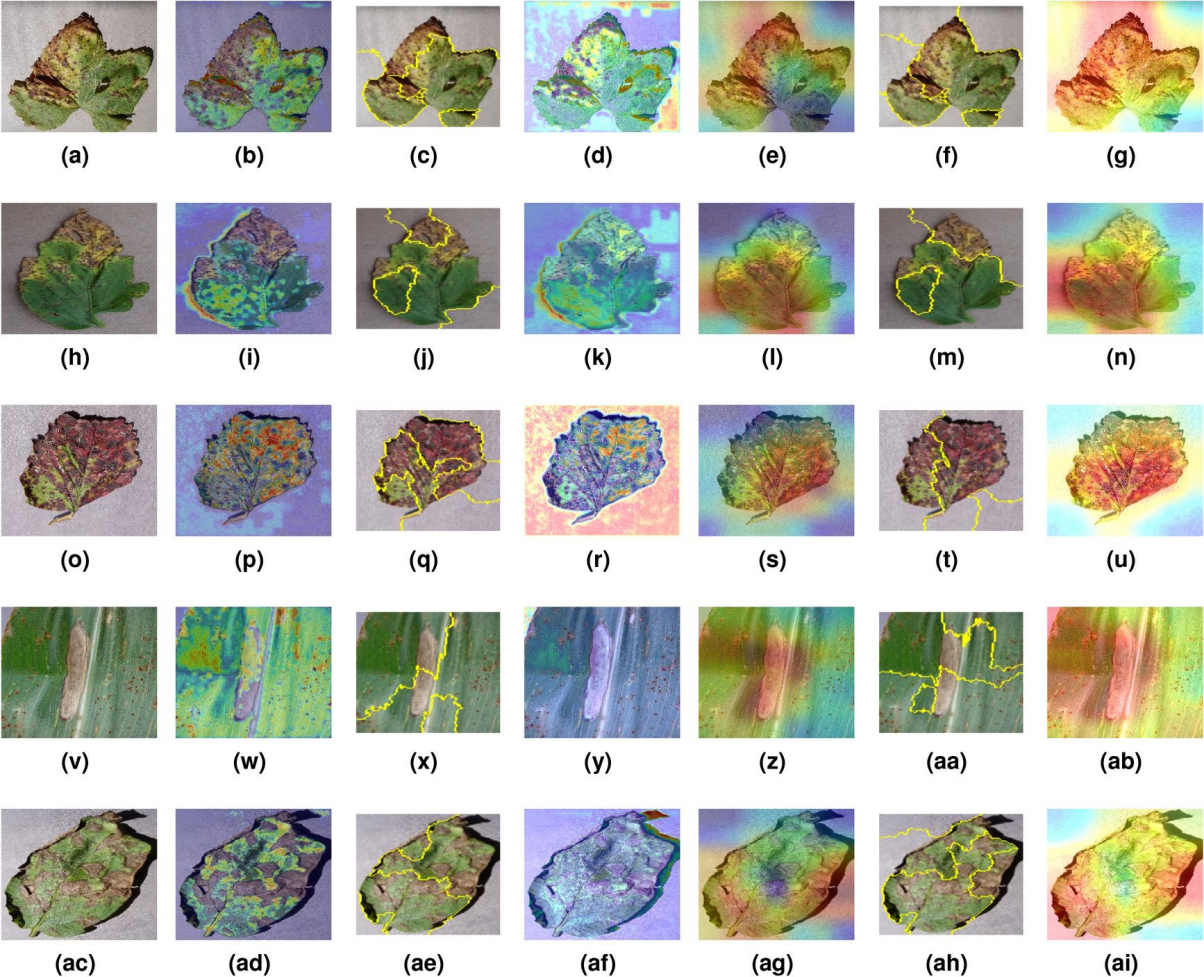
Visual representation of *Grad-CAM*, *LIME* and *Grad-CAM++* results on input images (1st column) for *Path1* (2nd, 3rd and 4th column) and *Path2* (5th, 6th and 7th column). Input images belong to classes: (**a**) *Grape Leaf Blight*, (**h**) *Tomato Bacterial Spot*, (**o**) *Strawberry Leaf Scorch*, (**v**) *Corn Maize Northern Blight*, and (**ac**) *Potato Early Blight*; (**b**), (i), (**p**), (**w**), and (ad) are the output of *Path1* using *Grad-CAM* for the corresponding classes; (c), (j), (q), (x), and (ae) are the output of *Path1* using *LIME* for the corresponding classes; (**d**), (**k**), (**r**), (**y**), and (af) are the output of *Path1* using *Grad-CAM++* for the corresponding classes; (**y**), (l), (**s**), (**z**), and (**ag**) are the output of *Path2* using *Grad-CAM* for the corresponding classes; (**f**), (**m**), (**t**), (**aa**), and (**ah**) are the output of *Path2* using *LIME* for the corresponding classes; (**g**), (**n**), (**u**), (**ab**), and (**ai**) are the output of *Path2* using *Grad-CAM++* for the corresponding classes.

### Interpretability for explaining the proposed *Mob-Res* architecture

Despite their impressive accuracy, *AI* models are not often transparent in their decision-making process, making them complex black-box systems that are challenging to interpret^31^. Interpretability is crucial in machine learning as it helps humans understand the reasoning of a model. This is particularly beneficial in scenarios where understanding the reasoning behind a decision is just as important as the decision itself. To assess the interpretability of our model, we employed *Grad-CAM* visualization^13^ on both *Path1* (residual blocks) and *Path2* (*MobileNetV2* feature extractor) of our *Mob-Res* model. Figure 8 shows the *Grad-CAM* results for both *Path1* and *Path2*, highlighting the corresponding regions of interest on the leaves. This method effectively highlighted the regions in the images most indicative of disease, such as the blight on grape leaves shown in Fig. 8f and the rusty areas on corn leaves depicted in Fig. 8n. These visualizations offer valuable insights into how the model makes predictions, revealing which features are emphasized when identifying diseases. They also expose where the model may make classification errors. For instance, when classifying *Strawberry Leaf Scorch* disease in Fig. 8m, *Path2*, which uses the *MobileNetV2* feature extractor, strays from the actual disease-affected regions. Similarly, for *Potato Early Blight* in Fig. 8o, *Path2* slightly identifies the area outside the leaf as a decisive feature. However, *Path1*, which uses residual blocks, mitigates these errors. In Fig. 8j, *Path1* correctly focuses on regions that closely correspond to the actual diseased areas for *Potato Early Blight*. These examples highlight that the hybrid *Mob-Res* model is less prone to errors than its individual components.

Incorporating *LIME*^13^ and *Grad-CAM++*^24^ provided further granularity in interpretability. Fig. 8c, 8j, 8q, 8x and 8ae demonstrate *LIME* results for *Path1*, while Fig. 8f, 8m, 8t, 8aa display results for *Path2*. *LIME* helped identify superpixels corresponding to diseased regions, but occasionally misfocused on irrelevant areas, such as in Strawberry Leaf Scorch (8q), where *LIME* for *Path1* highlighted parts of the leaf background. Also, in Potato Early Blight, *LIME* for *Path1* (8ae) correctly highlighted the blighted regions, offering clearer interpretability. *Grad-CAM++* refined the localization of important regions even further, especially in more detailed cases. For instance, Fig. 8d, 8k, 8r, 8y and 8af show the output of *Grad-CAM++* for *Path1* and Fig. 8g, 8n, 8u, 8ab and 8ai show the output of *Grad-CAM++* for *Path2*, providing better focus on specific blight spots, as seen in Corn Maize Northern Blight (Fig. 8ai). However, *Grad-CAM++* for *Path2*, as in Tomato Bacterial Spot (Fig. 8n), occasionally strayed from the disease-affected regions, indicating challenges in feature extraction precision for certain diseases. These visualizations offer valuable insights into how the model makes predictions and which features it emphasizes when identifying diseases. They also expose areas where the model may make classification errors. For instance, in Strawberry Leaf Scorch (Fig. 8r), *Path1* misfocused on the leaf background, while *Path2* provided a more accurate representation. Similarly, in Potato Early Blight (Fig. 8ai), *Path2* using *Grad-CAM++* closely focused on the diseased areas, mitigating errors observed in *Path1* (Fig. 8af).

Overall, the combination of *Grad-CAM*, *LIME*, and *Grad-CAM++* demonstrates the superior interpretability of our hybrid *Mob-Res* model, revealing that *Path2* generally exhibits more precise focus on diseased regions compared to *Path1*. This is corroborated by the model’s classification performance, where *Mob-Res*, with an accuracy of 99.45%, outperforms both *MobileNetV2* and residual blocks alone by 1.09% and 4.32% on Plant Disease Expert, as shown in Table 2. By integrating multiple interpretability approaches, we provide a comprehensive understanding of decision-making of *Mob-Res*, ensuring greater transparency and reliability for real-world plant disease diagnosis.

## Conclusion

In this work, we have introduced a novel deep learning architecture, *Mob-Res*, aimed at enhancing the accuracy of plant disease classification through a parallel-based *CNN* structure. To rigorously evaluate the generalization and adaptability of *Mob-Res*, we employed a comprehensive training strategy using both intra-dataset and cross-dataset configurations on *Plant Disease Expert* and *PlantVillage*. The model achieved an impressive accuracy of 99.47% on *PlantVillage*, outperforming several recent state-of-the-art works in the literature, demonstrating its ability to transfer knowledge effectively between datasets of varying complexity. A significant advantage of *Mob-Res* lies in its incorporation of the *Grad-CAM* technique, which significantly enhances the interpretability of the model. This method generates heatmaps that visually explain which areas of an input image most influence the decision-making process. By highlighting the regions most indicative of disease, *Grad-CAM* allows users to understand which features are being leveraged for predictions. This interpretability is crucial for validating the internal operations of the model, ensuring its real-world reliability. The proposed *Mob-Res*, with merely 3.51 M parameters, combines high accuracy with a lightweight design, making it well-suited for mobile-based agricultural disease diagnosis in resource-limited environments. This positions *Mob-Res* as an effective tool for real-time plant disease diagnostics in field settings. For future work, we aim to improve model generalizability by incorporating real-field data across seasons and growth stages. Factors like seasonal variations, crop age, and leaf damage can influence disease patterns, affecting model performance. Since the *Plant Disease Expert* and *PlantVillage* datasets do not explicitly account for these variations, integrating the considered factors in real-field data will enhance *Mob-Res*’s robustness and practical applicability in agricultural diagnostics.

## Data Availability

In this study, we utilized the *Plant Disease Expert* and *PlantVillage* datasets to train the models, with the widely used *PlantVillage* dataset serving as a benchmark for comparison against state-of-the-art models. The *Plant Disease Expert* dataset is publicly available on Kaggle at https://www.kaggle.com/datasets/sadmansakibmahi/plant-disease-expert. The *PlantVillage* dataset, a widely used benchmark for plant disease classification, can be accessed at https://github.com/spMohanty/PlantVillage-Dataset. Both datasets, as detailed in Section 3.1, are openly accessible to ensure research reproducibility. Additionally to showcase robustness of the model on field dataset, we have used a benchmark *Sugarcane Leaf Disease Dataset* as mentioned in Section Experiments on field dataset which can be accessed at https://data.mendeley.com/datasets/9424skmnrk/1.
